# First case report of fatal *Nocardia nova* infection in yellow-bibbed lory (*Lorius chlorocercus*) identified by multilocus sequencing

**DOI:** 10.1186/s12917-018-1764-x

**Published:** 2019-01-03

**Authors:** Sarah M. Churgin, Jade L. L. Teng, Jeremy H. P. Ho, Russell Graydon, Paolo Martelli, Foo Khong Lee, Suk-Wai Hui, Jordan Y. H. Fong, Susanna K. P. Lau, Patrick C. Y. Woo

**Affiliations:** 1 Ocean Park Corporation, Hong Kong, China; 2 Department of Microbiology, The University of Hong Kong, Hong Kong, China; 3 State Key Laboratory of Emerging Infectious Diseases, Hong Kong, China; 4 Research Centre of Infection and Immunology, The University of Hong Kong, Hong Kong, China; 5 Carol Yu Centre for Infection, The University of Hong Kong, Hong Kong, China; 6 Agriculture, Fisheries and Conservation Department, Government of the Hong Kong Special Administrative Region, Hong Kong, China; 7 Collaborative Innovation Center for Diagnosis and Treatment of Infectious Diseases, The University of Hong Kong, Hong Kong, China

**Keywords:** *Nocardia nova*, Infection, Yellow-bibbed lory, Multilocus, Sequencing

## Abstract

**Background:**

Nocardiosis is often a multi-systemic disease in humans and other mammals. Nocardiosis in birds is uncommon. Laboratory identification of *Nocardia* to the species level is difficult by traditional phenotypic methods based on biochemical reactions and hydrolysis tests, and is most accurately performed by sequencing multiple gene targets.

**Case presentation:**

We report the first case of fatal *Nocardia nova* infection in a yellow-bibbed lory nestling in an oceanarium diagnosed by multilocus sequencing. Necropsy examination showed effacement of normal sternal musculature with yellowish, firm aberrant material, and diffuse infiltration of the lungs with nodular, tan to yellow foci. Histologically, severe granulomatous inflammation with marked necrosis was observed in the lung, spleen and sternal musculature. Fine, sometimes Gram-positive, 0.5–1 μm wide, branching and beaded filamentous organisms were visible within the lesions. They were acid-fast on Fite-Faraco stain. Tissue samples obtained from the sternum, liver, right lung and right kidney recovered *Nocardia* species. Sequencing of four gene loci and phylogenetic analysis of concatenated (*gyrB*-16S-*secA1*-*hsp65*) sequences revealed that the isolate was *N. nova*.

**Conclusions:**

We report the first case of *N. nova* infection in yellow-bibbed lorry (*Lorius chlorocercus*). The present case is the first one of which the species identity of the isolate was determined by multilocus sequencing. Molecular diagnosis is important for identifying the *Nocardia* to species level and understanding the epidemiology of nocardiosis in birds.

## Background

*Nocardia* is a genus of aerobic non-sporulating Gram-positive branching filamentous rods that are partially acid-fast. *Nocardia* species are environmental saprophytes found in soil rich in organic matter. Nocardiosis is often a multi-systemic disease in humans and other mammals. Nocardiosis in birds is uncommon, with only around a dozen reports in the English literature [1–13]. Currently, there are more than 90 recognized *Nocardia* species. Laboratory identification of *Nocardia* to the species level is difficult by traditional phenotypic methods based on biochemical reactions and hydrolysis tests, and is most accurately performed by sequencing multiple gene targets [14]. In the present article, we report the first case of fatal *Nocardia nova* infection in yellow-bibbed lory (*Lorius chlorocercus*) identified by multilocus sequencing.

## Case presentation

A 45-day-old, captive-born, parent-reared yellow-bibbed lory was found dead in September 2017. The chick, which was hatched and raised in a semi-outdoor enclosure at an oceanarium in Hong Kong, had no prior history of medical problems and had previously appeared normal per animal trainers. It had been seen moving about in the nest box a few hours prior to death.

**Fig. 1:**
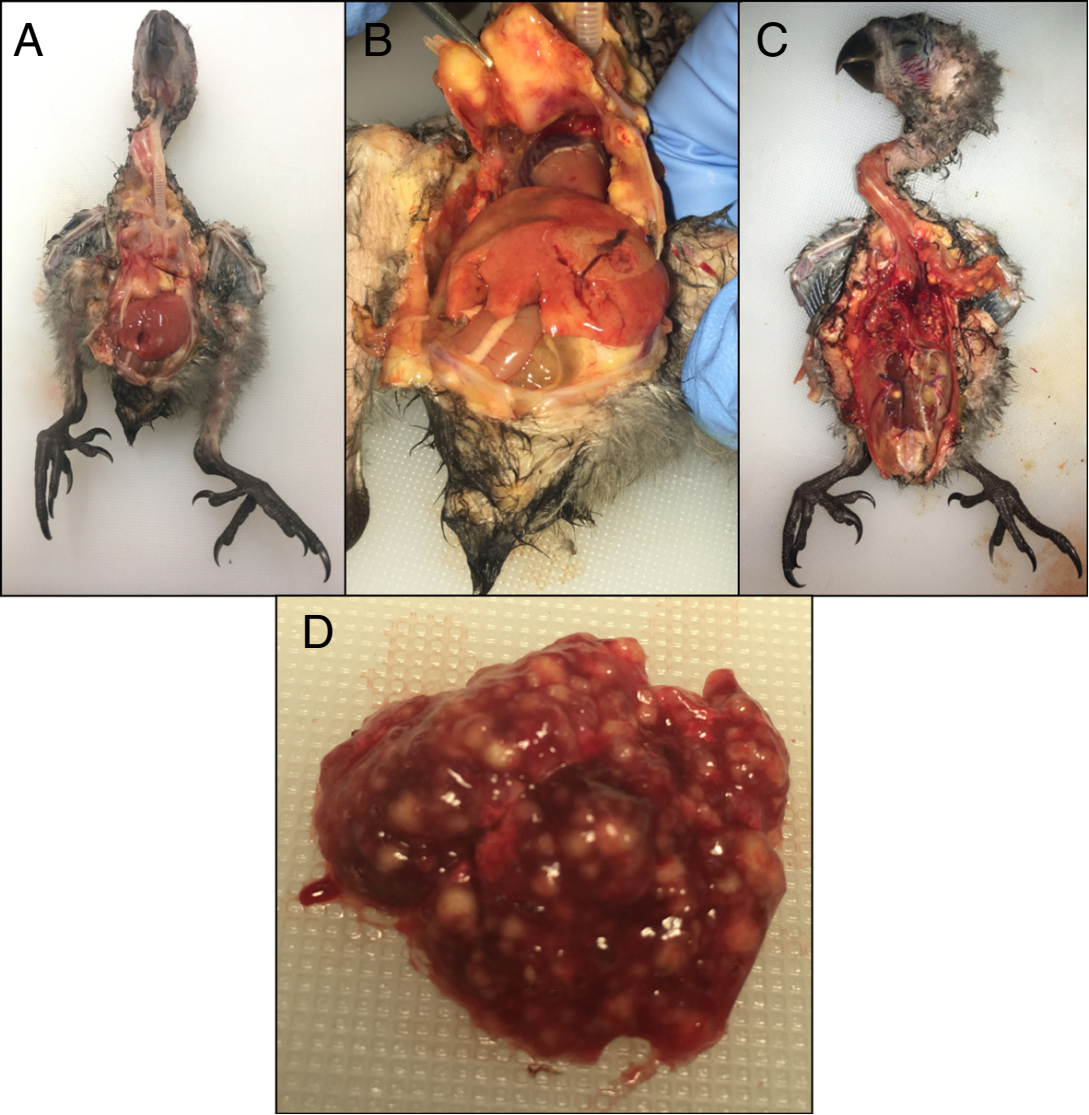
Necropsy findings of the yellow-bibbed lory **a** Abnormal yellowish tissue overlying the keel (sternum) prior to removing other organs. **b** The keel being reflected away, demonstrating the firm, adhered, yellowish material with hemorrhagic areas on the inner (serosal) surface of the keel. **c** Markedly abnormal lungs filled with tan to yellow foci and abnormal kidneys with a yellowish hue to the cranial pole of the left kidney and a yellow focus in the mid region of the right kidney. **d** Close-up of the abnormal lungs filled with irregular tan to yellow abscesses

External examination of the carcass revealed a nestling in good body condition with no gross evidence of post-mortem decomposition. There was polydactyly of digit 1 of the right foot. Internal exam revealed multiple abnormalities (Fig. 1). On both the external and serosal surfaces of the sternum, the normal musculature was replaced by diffuse, firm, and glistening yellowish tissue admixed with hemorrhage. The lungs were diffusely abnormal with much of the normal tissue replaced by nodular, irregular, and tan to yellow foci throughout both lobes. Lung tissue sank in formalin. The liver was enlarged and friable. There was a tan to yellow and creamy focus on the right kidney and the cranial pole of the left kidney was diffusely pale.

**Fig. 2:**
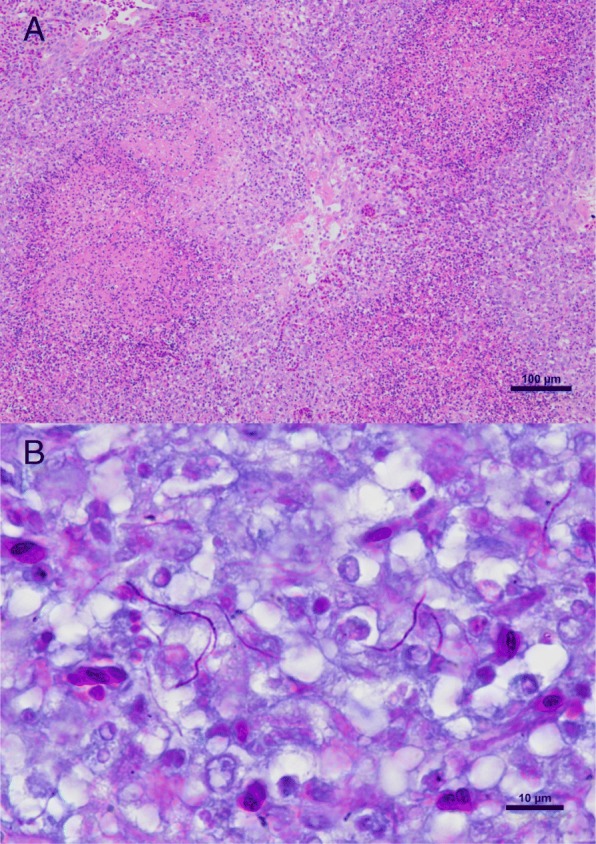
Histological sections of the lung. **a** Multiple granulomas with central necrosis. The granulomatous inflammation is characterized by macrophages, lymphocytes, fewer heterophils and occasional multinucleated giant cells. Hematoxylin and eosin stain (Bar = 100 μm). **b** Fine, beaded filamentous acid-fast organisms are visible in parts of the lesions. Fite-Faraco stain (Bar = 10 μm)

Histologically, severe granulomatous inflammation with marked necrosis was observed in the lung, spleen and sternal musculature destroying the normal architecture of the tissues (Fig. 2a). Fine, sometimes Gram-positive, 0.5–1 μm wide, branching and beaded filamentous organisms were visible in some locations within the lesions. These organisms were readily visualized by Grocott’s methenamine silver stain and were acid-fast on Fite-Faraco stain, but not on Ziehl-Neelsen stain (Fig. 2b). The Bursa of Fabricius was also examined and found to be normal histologically.

**Fig. 3:**
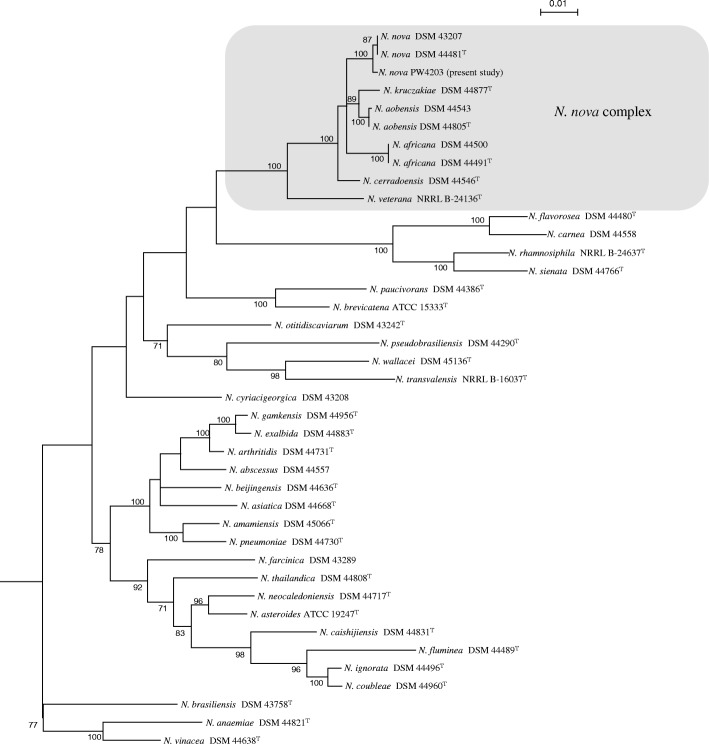
Phylogenetic tree showing the relationship of the *N. nova* strain isolated from the yellow-bibbed lory and closely related *Nocardia* species. The tree was inferred from the concatenated *gyrB*-16S-*secA1*-*hsp65* sequences by the neighbor-joining method using MEGA 6.0 [16]. One thousand seven hundred seventy six nucleotide positions were included in the analysis. The tree was rooted using the corresponding sequence of *Gordonia bronchialis* DSM 43247. Bootstrap values were calculated from 1000 trees and values below 70% are not shown. The scale bar represents 0.01 substitutions per nucleotide position. Names are given as cited in the GenBank database

Tissue samples obtained from the sternum, liver, right lung and right kidney yielded tiny colonies on 5% horse blood agar after 2 days of incubation at 37 °C with 5% CO_2_*.* The bacterium was Gram-positive, non-sporulating, beaded rods that formed branching filaments. It was modified Ziehl-Neelsen stain positive and catalase-positive, compatible with a *Nocardia* species. Matrix-assisted laser desorption ionization time-of-flight mass spectrometry analysis, using sample preparation method previously described [15] and Bruker Biotyper with the database V.6.0.0.0, identified the bacterium as *N. nova* or *N. asteroides* (both species with score > 2.0). Amplification of four gene loci, including β-subunit A of type II DNA topoisomerase (*gyrB*), 16S rRNA, subunit A of SecA preprotein translocase (*secA1*) and 65-kDa heat shock protein (*hsp65*), was performed using primers and PCR conditions as described previously [14]. A 481-bp, 462-bp, 445-bp and 401-bp fragment in the *gyrB*, 16S rRNA, *secA1* and *hsp65* genes were amplified and sequenced respectively using extracted DNA from the isolate. Gene sequences of the isolate were compared to those of other known *Nocardia* species using ClustalW by multiple sequence alignment, and the results showed that the isolate was most closely related to *N. nova* strains belonging to the *N. nova* complex, which included *N. nova* DSM43207 and *N. nova* DSM44481^T^, sharing 99.6, 100, 100 and 99.5% nucleotide identities on the *gyrB*, 16S rRNA, *secA1* and *hsp65* genes respectively. Phylogenetic tree was constructed based on the concatenated (*gyrB-16S-secA1-hsp65*) sequences using neighbor-joining method by MEGA 6.0 [16], and the results confirmed that the isolate was a strain of *N. nova* (Fig. 3). Also, an attempt was made to identify the source of the bacterium from various food and soil samples in the chick’s enclosure but all cultures were negative for *Nocardia*.

## Discussion and conclusions

We report the first case of *N. nova* infection in yellow-bibbed lory. Nocardiosis is uncommonly reported in birds, with only 13 reports in the English literature [1–13]. Two of the 13 reports involved outbreaks in 67 domestic pigeons and 8 black crakes respectively [3, 4], whereas the others were isolated cases. Among all the cases/outbreaks reported, the diagnosis in most of them was confirmed by histopathology, with the characteristic Gram-positive bead-rod observed invading the tissues. In one of the reports, in situ hybridization was performed on the histological section [2]. Similar to the present case, multiple organs of the birds were infected in 6 reports [1, 3–5, 7, 12]. In the 13 reports, birds of 10 different families have been involved. Parrots were infected in four reports, including nestling Derbyan parrot (*Psittacula derbiana*), rainbow lorikeet (*Trichoglossus moluccanus*), red-lored amazon parrot (*Amazona autumnalis*) and Pesquet’s parrot (*Psittrichas fulgidus*) [1, 5, 6, 9]. The present report is the first one which a yellow-bibbed lory (*Lorius chlorocercus*, a parrot in the family *Loriidae*) was infected. Similar to the previous two outbreaks [3, 4], the exact source of the infection is still obscure in the present study but is presumed to be environmental. Testing of food and soil samples was negative for *Nocardia* growth, but exhaustive sampling of other components in the habitat such as vegetation was not performed. Because the enclosure was semi-outdoors and the diagnosis of nocardiosis was not made until several days after death, further environmental sampling would have been poorly reflective of the conditions that led to infection. Chicks at this young age are still fed by the parents via regurgitated crop contents, so the infectious agent may have been passed to the nestling during this process.

It is unclear why this young chick succumbed to an opportunistic, severe, systemic infection of this nature. There was no evidence of immunosuppression or viral co-infection histologically, and the animal had been eating well with good body condition. However, at the chick’s young age, full immunocompetence would not be expected yet and this may have played a role in pathogenesis. It is also interesting to note the polydactyly of one digit, an unusual anomaly that suggests dysfunctional or disrupted embryonic development. It is possible that other undetected anomalies may have predisposed this chick to opportunistic infection. No further cases of nocardiosis have been diagnosed in yellow-bibbed lories or other species at this facility.

Molecular diagnosis is important for identifying the *Nocardia* to species level and understanding the epidemiology of nocardiosis in birds. Among the 13 reports, the bacteria were isolated in six [3, 4, 6, 8, 10, 11]. In all of them, the strains were identified by phenotypic tests only, mainly by morphology and their staining properties. Although three of the isolates were claimed to be identified as *N. asteroides*, the detailed identification methods were not described in two of them [6, 8, 10]. For the isolate that was identified as *N. nova*, it was distinguished from *N. asteriodes* through its acrylsulfatase activity and its susceptibility to cefamandol and erythromycin [4]. None of the isolates was identified by DNA sequencing. It has been shown that unlike most other bacterial species, sequencing one gene locus is insufficient to identify *Nocardia* to the species level [14]. On the other hand, amplification and sequencing four gene loci is essential for species identification, and the present case is in fact the first one of which the isolate was identified to species level by multilocus sequencing.

## Abbreviations

16S rRNA: 16S ribosomal RNA
*gyrB*: β-subunit A of type II DNA topoisomerase
*hsp65*: 65-kDa heat shock protein
*secA1*: subunit A of SecA preprotein translocase
